# Emergence and Embodiment in Economic Modeling

**DOI:** 10.3389/fpsyg.2022.814844

**Published:** 2022-05-06

**Authors:** Shabnam Mousavi, Shyam Sunder

**Affiliations:** ^1^Center for the History of Emotions, Max Planck Institute for Human Development, Berlin, Germany; ^2^School of Management, Yale University, New Haven, CT, United States

**Keywords:** embodiment, emergence, modeling behavior, three tiers, optimization

## Abstract

Exploratory ventures outside the established disciplinary boundaries can yield added insights and explanatory power. Imposing cognitive limitations on human logical reasoning ability (bounded rationality) is a well-known case in point. Extending cognition to parts of body outside the brain, and to environment outside the body is another. In contrast, the present article takes a constructive approach, also in an exploratory spirit. For the sake of exposition, we consider three tiered realms of scientific inquiry: physical or inanimate, biological or animate, and socio-psychological or sentient. In this three-tier framework, we explore the extent of gains in modeling human action within the confines of physical principles such as optimization. In this exercise, concepts of complexity and emergence account for the absence of analytically derivable mapping from micro or finer grain phenomena to macro or coarser grain phenomena. A general notion of embodiment captures the inclusion of a more expansive range of explanatory factors in modeling and understanding a given phenomenon. Emergence and embodiment play complementary roles in exploration of human behavior.

## Introduction

Conceptual foundations of cognitive science of human (and animal) behavior rest on two assumptions to locate cognition in brain: objects in the environment being represented as symbols in the brain, and the brain functioning as a computer to process these symbols. During the past half-a-century it has been suggested that parts of the body outside the brain, as well as the environment outside the body, play a role in cognition. Furthermore, evidence points to the possibility of this dependence of cognition on extra-cranial parts of the body and external environment being structural, and not merely causal (Viale, 2012, 2014; Gallese and Cuccio, 2015; Varela et al., 2017; Gallagher, 2020; Vincini and Gallagher, 2021). The conceptual extensions beyond the traditional confines of cognitive science (representation and computation inside the brain) to other parts of the body and to the larger environment have taken several partially overlapping approaches (Wilson, 2002) under the labels of embodied, embedded, extended and enacted (collectively referred to as the 4Es in Newen et al., 2018), as well as distributed (Hutchins, 1995) and situated cognition (Gallagher and Varga, 2020). These developments either reject or reconfigure traditional cognitivism (Menary, 2010).

In this article, we ignore the distinctions among the diverse arguments and theories listed at the end of the paragraph above, and use “embodiment” as a common label for them in the meaning given therein. While obviously unsatisfactory for discussion of cognition, it would suffice for our objective of exploring the complementary role of embodiment and emergence in modeling human behavior. We use these two terms in the following intended meanings. Manifested within as well as between tiers, emergence is the phenomenon of complex interplay among individual (or finer grain) elements giving rise to distinct coarser grain or aggregate level phenomena with properties absent in the parts. Embodiment implies that cognition is not limited to the brain, but includes parts of the body outside the brain, as well as elements of the environment outside the body and social interactions.

Emergence appears often in analyses of markets (Gode and Sunder, 1993; Sunder, 2006; Smith, 2008, 2009, 2010), and in complexity economics (a term coined by Doyne Farmer^1^). Viewing mental abilities (cognition) as emergent phenomena has precedent in cognitive science, development psychology and artificial intelligence research for many decades (Clark, 1997; McClelland, 2010). While institutions' role as location and enablers of emergence that extend agents' own minimal cognition is compatible with embodied cognition (Gilbert and Terna, 2000; Gallagher et al., 2019), on the whole, the idea of embodiment is relatively newer in economics than emergence. Moreover, with the exception of cognitive economy (Rosch, 1978), which is instantiated through embodiment, the mainstream cognitive economics, like cognitive psychology, is primarily focused on what is in the mind (Kimball, 2015). Overall, work that combines embodiment with emergence remains scarce (for philosophical instances see Garrison, 2022; Ryan, 2022).

It may be useful to start with a thumbnail sketch of developments in modeling human behavior in economics. Axiomatization of choice by von Neumann and Morgenstern (1944) was expanded to include subjective expected utility by Savage (1954). To date, expected utility theory (EUT) remains the corner stone of economic analysis of human behavior and the economic theory of choice (on its empirical failure, see Friedman et al., 2014). Given the ubiquity of methodological individualism and the concomitant psychological foundations of microeconomics, rise of cognitive science in the middle of the twentieth century led to a behavioral critique of economic theory. It was rooted in the discrepancies between the psychological assumptions about human decision-making on one hand and observed human cognitive abilities on the other. By incorporating known limitations of human cognitive abilities, bounded rationality was introduced as a revised framework for economics (and related aspects of other social sciences) to reshape the classical microeconomic approach, which had remained rooted in unbounded cognitive abilities (Simon, 1957). In other words, bounded rationality sought to improve the explanatory power of economic models using the accepted cognitive science framework, referred to as cognitivism, that keeps cognition firmly located in the brain (Mousavi and Garrison, 2003). Section Economic Models Keep Cognition in the Brain expands on this and provides the larger context of this development. We believe that the inclusion of the roles of extra-cranial and environmental phenomena in the expanded conceptual scheme of embodied cognition call for revisiting its implications for the use of economic theory to organize observed phenomena. Extend the current economic theory in this manner promises to produce better explanatory power and newer insights. However, that ambitious task is beyond the scope of this article.

Instead of expanding outside the traditional boundaries, this article takes a confining approach to the study of human behavior. More specifically, we take a few steps back, away from higher faculties such as intention and cognition, and even from evolutionary and other biological attributes. Limiting our exploration within the boundaries of inanimate existence, we examine just how much can be understood and what can be gained from modeling human action by framing it only in physical terms. This is not a reductionist approach; we remain fully cognizant of the aspects of behavior that cannot be understood without biology and socio-psychology. What we want to emphasize is to keep the interpretability of principles of every discipline within its confines, while also allowing their use as structuring tools on the outside. An example of a powerful organizing tool is in the domain of physical sciences (Sunder, 2006). To illustrate, we use the principle of least action from physics to reconfigure some extant models, and to compare that to a fully physical representation of the same phenomena. This reconfiguration does not enhance the explanatory power; instead, it helps address the well-known criticism of using optimization to model human behavior in economics on grounds of limited cognition. Once the cognitive or biological element is not a part of the model, questions about the applicability of optimization to modeling human behavior lose their relevance. Distinguishing reality from models, our physics-first approach is implemented in a three-tier framework. It is introduced in the next section, where the prevalence of cognitivism in modeling behavior is discussed. Section Where We Start Modeling Matters focuses on the shared physical existence of animate and inanimate worlds, where optimization is a powerful explanatory principle. The takeaway is not that all phenomena can be reduced to their physical existence. Section Causality Is Also in Tandem with Modeling Direction discusses causality. Section Methodological Individualism: Trouble No More argues that starting the modeling of human behavior from physical domain recasts longstanding concerns about methodological individualism in a new light by adopting an approach that keeps principles of each discipline within its boundaries. Section Embodiment Is an Outward Approach portrays embodiment as an outward perspective, and Section Concluding Remarks concludes.

## Economic Models Confine Cognition in the Brain

Human mind is thought to operate in a neurobiological brain in the physical world and with culture in the social world. Its existence comprises multiple discrete interacting layers (Anderson, 2007; Frégnac and Laurent, 2014). Cognitive capabilities are not only traced in the activities of neurons in the brain (e.g., fMRI), but also in muscle memory that produces embodied automatic behavior (e.g., athletic training). Embodied cognition offers an alternative framework that admits cognition to extend outside the brain. Such extension is largely missing in economics. More than a century of scientizing of economics in the image of mathematics and physics has focused on refining reverse-engineered models of human behavior from observations. By and large, neuroscientists are actively engaged in similar activities (on neuroeconomics, see McCabe, 2008). Along the way, behavioral economics rose by focusing on imperfections of such models, and attempted to increase explanatory power by drawing on evidence from cognitive psychology and other disciplines. These efforts have been criticized for concentrating largely on blind alleys of unusual or contrived experiences covered neither by human evolution (Aumann, 2019) nor life experiences of most individuals. In the terminology of behavioral economics, anomaly is a generic label for observations that deviate from the predictions of the expected utility theory about individual behavior. Complications, messier mathematics, unobservability of explanatory constructs and the consequent decline in intuitive appeal are the main challenges to increasing the explanatory power sought from accounting for an ever-expanding list of anomalies.

At the aggregate level, either average behavior or emergence from complex interactions among micro-behavior can become salient, making it more observable and easier to theorize about. But for making economic policy and supporting recommendations, ease of analysis is not without its own disadvantages. Applying equilibrium and optimization concepts from physics beyond its traditional inanimate realm—say, to sentient phenomena—has important consequences that merit scrutiny. Do we need to completely abandon optimization (or in general, tools of analysis in physics) to produce sensible results? We suggest a technical modification to the use of methods, instead.

**Figure 1:**
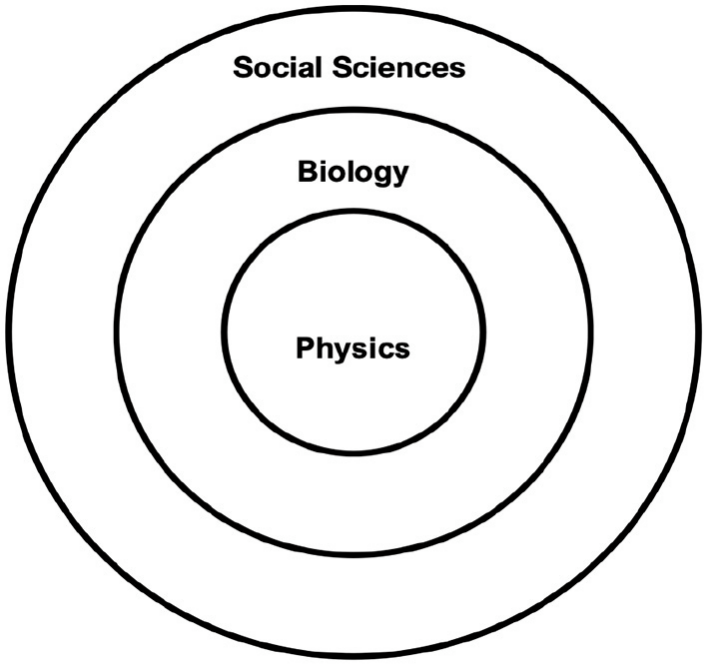
Earth metaphor for the three-tier framework for modeling human action. The familiar direction is from the crust inwards and we explore the outward direction (Art by Anoush Kheirandish).

In an attempt to revisit methods of modeling human behavior, as an instrument, we developed a simple three-tier framework (Mousavi and Sunder, 2019, 2020). Consider organizing observed phenomena in three tiers using metaphor of crust, mantle and core in planet earth: human actions are manifested in the crust, biology in the mantle, and physics in the core (see Figure 1). While subject matter of physics concerns the universe of inanimate matter and energy, including the smallest of particles, human behavior encompasses sentient phenomena, with biological perspective situated in between the two.

Note that the extant method, by and large, takes an inward approach to modeling human behavior: from crust to mantle to the core in the earth metaphor in Figure 1. For example, efforts to model altruism start with social-psychological attributes such as utility, reciprocity, empathy, and identity. Appeal to principles of biological evolution may contribute some additional explanatory power through survival of the species. Only then might the modeler resort to abstract mathematical apparatus from physics.

The applicability of optimization to human choice behavior has long been debated. The main defense lies in good performance and lack of better alternative (Stigler and Becker, 1977). In Grether and Plott's words:

The fact that preference theory and related theories of optimization are subject to exception does not mean that they should be discarded. No alternative theory currently available appears to be capable of covering the same extremely broad range of phenomena. In a sense the exception [preference reversal] is an important discovery, as it stands as an answer to those who would charge that preference theory is circular and/or without empirical content. It also stands as a challenge to theorists who may attempt to modify the theory to account for this exception without simultaneously making the theory vacuous (Grether and Plott 1979, p. 629).

Notably, acknowledgment of cognitive limits in models of bounded rationality has not led to discarding optimization as a powerful tool for analysis. Models under bounded rationality paradigm also construct paths of action of satisficing agents that are optimal subject to their cognitive and procedural attributes. Moreover, cognition remains firmly located in the brain, in both bounded as well as unbounded paradigms.

Is optimization principle a legitimate tool for analyzing human behavior? We believe it can be, as long as it is confined to analysis in the physical core shared by animate and inanimate existence. Therefore, optimization presents a meaningful frame for modeling human action as long as its implications remain within that core. Doing so only requires the modeler to focus on the shared properties of matter and energy in the universe first, before attending to the biological and social-psychological characteristics of the animate and sentient phenomena. In context of the earth metaphor in Figure 1, we suggest an outward approach to deployment of tools of analysis and to confine the interpretability of each set of principles to its respective tier. This implies using optimization for analyzing human behavior, but only to capture the components that might be shared with inanimate phenomena as elaborated in the next section.

## Where We Start Modeling Matters

Consider four examples from different domains, each presenting an action or end point of an action: (a) a jar filled with small smooth marbles, (b) the network of nerves among ganglia (nodes) of a nematode worm, (c) a baseball player running to catch a fly ball, and (d) iron filings on a plate in the force field surrounding a magnet. Now, let us explore how far can optimization takes us in organizing these four observed phenomena.

a) When a large jar full of small smooth marbles is shaken for a few seconds in a gravitational field, packing of its contents approaches a local optimum arrangement.
b) Connections among the ganglia in a nematode's nervous system are optimized to save wire:
At multiple hierarchical levels—brain, ganglion, and individual cell—physical placement of neural components appears consistent with a single, simple goal: minimize cost of connections among the components. The most dramatic instance of this “save wire” organizing principle is reported for adjacencies among ganglia in the nematode nervous system; among about 40,000,000 alternative layout orderings, the actual ganglion placement in fact requires the least total connection length. In addition, evidence supports a component placement optimization hypothesis for positioning of individual neurons in the nematode, and also for positioning of mammalian cortical areas (Cherniak 1986, p. 1).
c) Cognitive scientists have used data gathered from the field to model how animals and humans catch fly balls and other moving objects. They keep a constant angle of gaze on the ball above the horizon while moving toward the ball until catching it. If we take an outward approach to model this phenomenon (to catch a fly ball) a mere minimization of changes in the angle of gaze fixed at the ball would suffice. However, the inward approach consists of the following elements: (1) cognitive attribution of catching the object by deploying the cognitive ability to hold the gaze on a moving object against a noisy background; (2) biological attribution: evolution of capabilities for preys to evade predators and for predators to catch their preys; and (3) physics scheme: solving an optimization problem with the objective of minimizing the change in the angle of gaze—ideally, keeping the angle fixed (for a comprehensive overview of the phenomena, see Hamlin, 2017).
d) Orientation of the iron filings aligned with the direction of the magnetic field represent an optimal outcome albeit subject to approximation depending on the size of the filings, strength of the field, and friction with the supporting surface.

All four seemingly disparate phenomena discussed above exhibit presence of optimization at work. Our proposed outward approach offers alternative ways of organizing a given phenomenon at various levels. In what follows, first we organize items b (the nervous system of a simple worm), and c (catching a fly ball) by using the physical principle of least action (PLA) and remaining confined to physical attributes. We also use PLA to organize the inward modeling approach for the same two phenomena. By juxtaposing the resulting structures, we show how this exercise can produce a method for comparing modeling elements among different tiers (see Table 1). Second, we generalize our three-tier framework to organize scientific inquiry across fields of study (see Table 2).

**Table 1:** Using the principle of least action to model catching a fly ball and the nematode nervous system (Source: Mousavi and Sunder, 2021).

	**Method of modeling**	**WHAT: given variables**	**HOW: action element**	**Path of action**
To catch a fly ball	*Current Method: Inward approach with three-tiers*	Time a fly ball takes to reach ~1.5 m above ground	Use the evolutionary capacity of holding gaze on a moving object	A curved path, depending on when the angle of gaze is first fixed
	*Proposed method: In the first physics tier only*	Same as above	Keep a *fixed angle* of gaze (change = 0)	Same as above
Arrange nervous system network	*Current method: Inward approach in the second tier*	Location of ganglia in a combinatorial space	Economize the use of biological resources for connecting (ganglia)	A path of fiber connections with minimal length of connections
	*Proposed method: In the first physics tier only*	*Number of ganglia*	Minimize distance among ganglia and position them concurrently	Same as above

**Table 2:** Subject matter and principles in three domains of scientific inquiry (Source: Mousavi and Sunder, 2021).

**Domain**	**Animate**	**Animate-Inanimate**	**Inanimate**
Discipline	Social Sciences	Biology/Molecular Chem.	Physics/Chemistry
Subject matter	Person/group/institution	Large molecules/Cells/Organism/group	Matter and energy (detectable and dark)
Principles, concepts and terms	Theories of mind Perception and cognition Nature vs. nurture Demand and supply Behavior, labor, capital, trade, contract, judgment, personality, development State and society…	Evolution by natural selection (Matching) Longevity vs. reproduction Function of organs Anatomy and physiology DNA, RNA, cells, protein, life…	Least action Force fields Chemical binding Inertia and Symmetry Relativity Effort, flow, motion, time …
Shared features	Physical existence in all domains is subject to physical laws.		

The first exercise demonstrates that using a physics principle for organization does not imply that all elements of the observed phenomenon need to be only physical. Let us examine how one physics principle can be used to organize and compare a physics-only model with a biology-based description. The principle of least action structures an observed phenomenon with specifying three elements: (1) an action element that is the argument of optimization, (2) given or fixed element(s) that are not affected by the action but constrain it, and (3) a path of movement on which the action is realized. Table 1 lists the physical forms of these elements for catching of the fly ball and the nematode nervous system when the modeler remains confined in the physical core, as well as the biological (evolutionary) forms of the same element that are used in the familiar inward methods of modeling. Organized as such, comparison and connections among elements of modeling is straightforward. This can facilitate interdisciplinary communication and collaboration. Indeed, we consider our framework as a productive and generalizable method for detecting cross sections of scientific pursuits and initiating cross-disciplinary exchanges for virtually all fields of study.

The second exercise features thinking in terms of three tiers as a powerful tool that can be used for structuring not only modeling practices concerned with human behavior, but also a general view of scientific inquiries into the inanimate, animate and sentient existence. Our attempt to organize fields of study in this manner is summarized in Table 2.

Where we start modeling matters. Economists traditionally, and psychologists increasingly produce policy recommendations and intervention designs. We argued that social scientists in general take an inward approach to modeling human behavior. This means that they can easily ignore the eventual effect of physical structures at work. This inattention can be consequential in a large scale, especially when the modeling and observation methods are assumed to be neutral with respect to the outcome, or independent of each other. Scientific observation at large has a history and evolving structure:

The scientific observation of the organic world (including humans) went through three stages: first, intensive observation of very small samples (still pursued in primatology); second, statistical observation of large samples to extract averages (still used in much of social science); and third, observation of larger samples that focused on variability rather than erasing it with averages (striven by Darwin's insight that it is individual variability that drives evolution). All three modes of observation are still very much in use and often complement one another: for example, a puzzling statistical effect may need a more granular ethnographic study to discover the causal mechanisms at work^2^.

Physicists are wary of the observer's role, and statisticians' motto is: if you beat the data long enough, it will eventually confess. Social scientists regularly talk about scientific facts derived from data. Both the sequence and the limits are of particular consequence in using results from physics models to draw societal implications. We therefore propose a careful observation of the sequence in which scientific insights are gained, cognizant of the similarities and differences between social, biological, and physical phenomena as summarized in Table 3.

**Table 3:** Properties of different phenomena.

**Subject matter**	**Physical phenomenon**	**Biological and social phenomenon**
**Scientific inquiry**		
Observation effect	Yes	Yes
Principle universality	Yes	No
Method neutrality	No	No
Explanatory equivalence	Yes	No

Choice of starting point of modeling matters. What is and is not carried across the tiers of scientific inquiry also matters. Critiques of a physics-based approach to socio-economic phenomena can be recast by switching the direction of sequence of investigation from inward to outward. At a time when behavioral policymaking has spread far and wide in public (and private) sectors, it is refreshing to recount astrophysicist John Stewart's insights:

There is no longer [an] excuse for anyone to ignore the fact that human beings, on the average and at least in certain circumstances, obey mathematical rules resembling in a general way some of the primitive “laws” of physics. “Social physics” lies within the grasp of scholarship that is unprejudiced and truly modern. When we have found it, people will wonder at the blind opposition its first proponents encountered.

Meanwhile, let “social planners” beware! Water must be pumped to flow uphill, and natural tendencies in human relations cannot be combated and controlled by singing to them. The architect must accept and understand the law of gravity and the limitations of materials. The city or national planner likewise must adapt his studies to natural principles (Stewart, 1947, p. 485, emphasis added).

## Causality Is Also in Tandem with Modeling Direction

Just as it is customary in scientific practice to start analysis of action with attribution to most salient, immediate and proximate variables (an inward approach), it is not unusual to assume the arrow of causation and dynamics pointing in the opposite direction from such variables to observations, especially if the former carries an earlier time stamp. Consider this example from a textbook on biology for engineers on effort (cause) and flow (effect):

There are two basic kinds of variables that describe the action of a physical system. Effort variables are those things that cause an action to occur. Flow variables are the responses to effort variables, usually involving movement but not always. For the simple case of a running animal, the effort variable is the force required to propel the animal; the flow variable is the velocity of movement. Heat loss from that same animal, which is the flow variable, occurs in response to a temperature difference, an effort variable. Sexual attraction to an animal of the opposite sex (effort variable) can result in a wide range of activities, including copulating (a flow variable). Hunger (an effort variable) can result in feeding (a flow variable). Thus, there are a wide variety of causes and effects related to biological activity, and these can be thought about in terms of effort and flow variables, which tend to simplify the concepts of biological activities. For any activity of a biological organism or system, searching for the effort variable, the flow variable, and relationships between these two can make it easier to comprehend not only how and why the activity occurs, but also the intensity of the activity (Johnson, 1941, p. 32–33).

This effort-flow frame captures a wide range of phenomena across domains from force and acceleration in Newtonian mechanics to motivation and work in social sciences. Extending this form of framing generates amusing views. For example, framed in economic terms, the outcome of sustainability can be achieved by optimizing on the flow variables of consumption and reproduction: “Consumption and reproduction have been and remain the basic values of human societies. These two lie at the root of our moral codes. Even virtue is promoted with the promise of entitlement to more consumption in the future. Development, prosperity and welfare are euphemisms for higher consumption” (Sunder 2012, p. 1).

Economics is the most physical of the social sciences, and has directly adopted physical terminology such as equilibrium, friction, efficiency. However, economics is not alone in this. It does not require much effort to trace conceptual links also between physical laws and other social sciences and with humanities.

The “path of least resistance” as the underlying principle of inductive sociology was introduced more than a century ago (Giddings, 1906). The linguist Zipf (1949) built the “biosocial physics” theory of human behavior whose principle of minimum effort yields the eponymous law of frequency distribution anywhere from of words in a language to city populations. Zipf considered mind as a system of “mentation”, by analogy extended the philology of semantics in spoken language and cultural preconceptions to the structure of every human action. In the context of embodiment approach, psychologist Rosch proposed cognitive economy as the first of her two principles that govern how human being categorize their world of language, people, animals, vegetation, and just about everything else in order “to provide maximum information with least cognitive effort …” (Rosch 1978, p. 28). Similar analogical exercises have been undertaken with the concept of inertia that links effort-flow and capacity. Economist Bewley (1987, 2002) used inertia to formulate economist Knight's (1921/2006) notion of uncertainty. In general, for framing cognition inertia has long been considered as a fundamental law. In the words of Schiller (1846–1937):

Our curious result of this inertia, which deserves to rank among the fundamental laws of nature, is that when a discovery has finally won tardy recognition it is usually found to have been anticipated, often with cogent reasons and in great detail (Johnson 1941, p. 35).

This very phenomenon is dubbed as the knew-it all-along effect in contemporary literature. Finally, the Lagrange principle for probability with constraint that views physics in terms of energy and entropy, is used in socio-physics to frame a wide range of phenomena across social sciences: planned vs. spontaneous, collective vs. individual, law as right vs. wrong, or order vs. disorder; society as bondage vs. freedom; and economics as rational vs. chances (Chakrabarti et al., 2006).

Overall, the inward deployment of principles—from social-psychological to biological to physical properties—has generated a body of coherent models, partially generalizable theories, as well as numerous ongoing disagreements. In the next section, we argue that our outward-confined approach is not an effort to answer a major critique to this practice, namely methodological individualism, instead it is a new way of thinking and organizing the matter that addresses the problem at hand in a different light.

## Methodological Individualism: Trouble No More

Philosopher of science, Longino, takes issue with the general thrust of modeling behavior:

[T]he question [of behavioral sciences] is why people fall into one or the other of these categories, or fall into a particular range of a multiple-valued quantitative (more or less) trait. Behavioral sciences seek to answer this question. Even when the research methodology permits only correlations among behaviors and studied factors, it is intended ultimately to contribute to an understanding of the causes of behaviors. To ask about the causal influences on the expression of a trait in a population is already to be committed to an individualistic point of view….factors maybe genetic, hormonal, neurological, or environmental. The question for researchers is how these factors influence an individual's disposition to respond to situations in one way or another (Longino, 2019, p. 4).

Methodological individualism is a cornerstone of much economic thought of the recent century. Starting with individual (human being) as the primary unit of axiomatization, actions and analysis, economic theory derives and predicts outcomes and economic behavior of organizations, markets, and societies.

In economics, as in other social sciences, engineering approach to designing the parts to serve the functions of the whole and building the whole from the parts takes the form of methodological individualism. Even when the macro or coarser grain phenomena phenomenon is of primary interest, modeling starts with specifying attributes of the individual at micro level, where the “representative agent” manifests shared attributes. Macro outcomes of the model result from a constructivist process that derives properties of the collective from behavior of sophisticated individuals who demonstrate rationality in anticipation, learning, and goal-seeking. Reflecting on this common practice, economist Arrow highlights the social nature of all economic phenomena:

In the usual versions of economic theory… seems commonly to be assumed methodological individualism, that it is necessary to base all accounts of economic interaction on individual behavior… A specific version of this has invaded other social sciences, under the name of rational-actor models. … [There exists] explicit advocacy of methodological individualism among the Austrian school…[and] useful implications of methodological individualism for positive economics. It is usually thought that mainstream economics is the purest exemplar of methodological individualism [but]…. In fact, every economic model one can think of includes irreducibly social principles and concepts… social variables, not attached to particular individuals, [which] are essential in studying economy or any other social system… (Arrow 1994, p. 8).

Methodological individualism lies at the heart of choice theory and rests on two key psychological elements: preferences (whether static or adaptive) and choice of preferred alternative(s) from the individual's opportunity set specified by the environment. Preferences of an individual are a mapping from objects of choice to the real line, so each object is either more, or less or equally preferred to every other object under consideration. Preferences may be static, dependent on the state of the world, and may change over time according to a knowable law. Remarkably, if the law by which the preferences change is not knowable ex ante, anything goes, and they could not serve as a basis of a theory (for an engaging study, see Pastor-Bernier et al., 2017).

The rational-actor model encounters two hurdles in scaling up to social phenomena. As the number of agents increases, so do their opportunity sets, strategies, actions and interactions among them, rapidly rendering analysis of interactions intractable. Representative agents and other simplifying assumptions made to facilitate analysis add the risk of excluding important social dimensions. Second, when macro-level phenomena emerge from non-linear complex interactions among many parts, the properties of such outcomes may not be derivable and cannot be constructed from the micro-level properties.

Deployment of emergence and embodiment in tandem as modeling apparatus can be portrayed as follows. Emergence can be a tool for explaining social phenomenon that cannot be adequately captured by economic modeling of rational agents. Similarly, embodiment provides a perspective for cognitive psychology, and for behavioral sciences in general, to account for the context that may include the body of actors and their social interactions.

## Embodiment Is an Outward Approach

Embodiment literature, replete with irrelevance of optimization to understanding cognitive phenomena, is in conformity with our proposal to keep principles of each tier confined within. For example, when moving to the animate domain, evolutionary capacities can be understood through their viability not optimization:

One of the more interesting consequences of this shift from optimal selection to viability is that the precision and specificity of morphological or physiological traits, or of cognitive capacities, are entirely compatible with their apparent irrelevance to survival. To state this point in more positive terms, much of what an organism looks like and is “about” is completely *underdetermined* by the constraints of survival and reproduction. Thus, adaptation (in its classical sense), problem solving, simplicity in design, assimilation, external “steering” and many other explanatory notions based on considerations of parsimony not only fade into the background but must in fact be completely reassimilated into new kinds of explanatory concepts and conceptual metaphors (Varela et al. 2017, p. 196).

It is commonplace to think of technology—fire, hammer, knife, eyeglasses, car, and telephone as external devices developed by humans over the ages and put to use to make their life easier and better. In this everyday perspective, the evolution of humans is confined to what is covered by their skin. However, this perspective can be questioned at three levels. First, to the extent tools and technologies enhance the ability of our own bodies to perform various functions, the former can be viewed as evolutionary extensions of the later. For example, hammer could be seen as evolution of hand, and bicycle as evolution of legs, where evolution extends outside the body. This perspective includes inanimate objects created by humans as extensions of humans themselves, fusing them across the matter-energy vs. biology boundary line.

Second, without crossing the inanimate/animate boundary, humans like other organisms have large microbiomes added to their “human” genetic endowment. The two parts of the total genome have evolved together to a state where their independent separate existence as life forms may not be viable. No humans are known to survive the destruction of gut bacteria. This relationship makes it difficult to decide if the genome of billions of microorganisms that reside in the gut of every human being are or are not to be regarded as a “part” of the human body.

Third, is the case of human body and the structure of societies in which humans live—such as family groups including two, three or more generations with specialization of work by age and gender. These social structures themselves could be seen as extensions of human evolution in a non-trivial sense.

All three kinds of evolution—within biology, between biological and social, and between biological and physical worlds—have a long history. Persuasive arguments have been made about their co-evolution. Early stone tools and electronic computers today are not only results of human brain but also helped shape the brain that created them. Same could be said of co-evolving species in the animate world. Human body and mind and tools surely co-evolved with each other, as also with the structure of families and human societies. Human child, for example remains dependent for longer than any other animal, and development of tools and fire may have been related to the length of gestation of child rearing.

In sum, our examination of embodiment in the three-tier framework reveals the outward direction of deployment of scientific principles. It is in this manner that embodiment provides better understanding of human behavior as well as more powerful tools for describing and explaining observed phenomena.

## Concluding Remarks

Familiar approach to modeling economic behavior starts with specifying social-psychological preferences and goals to construct an objective function, specifying the opportunity set by constraints, and then seeking optimal choice of action from the set. For example, an effort to understand the price and availability of coffee may start with attributing preferences to consumers, production technology to producers, and opportunity sets to them both, before deriving price and allocations from a model that attributes maximization of their respective goals—utility of consumers and profit of producers arising from their sentient and conscious nature. We introduced a three-tiered framework with physics in the core, biology in the middle, and socio-psychology on the top tier. Our framework characterizes this familiar method of modeling as an inward approach that originates in the crust with the possibility of proceeding to the biological middle, and to formalize uses the tools and principles from the physical core.

We discussed that optimization is a superb organizing principle for modelers. It manifests in the physical phenomena most vividly, for example in mechanics, sound, light, electricity, magnetism, and elementary particles. We set out to explore an outward approach to deployment of scientific principles. This attempt was motivated by the idea that if photons do not need cognitive equipment to optimize their paths from a candle to a book, there is no reason to presume in modeling—as is in the inward approach—that all human behavior must necessarily arise from animate adaptive and cognitive faculties at its physical level. Moreover, emergence of social phenomena and their properties, when optimal, can be decoupled from what is derivable from individual parts of the system. We argued that through an outward-confined approach, our three-tier framework organizes physical, biological and social science principles, proposing a new and broader perspective on human behavior, sans reductionism. Viewing embodiment and emergence as exploration methods that deploy scientific principles in an outward direction highlights their tandem role in scientific inquiry, each enriching insights into human behavior.

## Author Contributions

Both authors listed have made a substantial, direct, and intellectual contribution to the work and approved it for publication.

## Funding

SM acknowledges the Original-isn't it? grant from the Volkswagen Foundation.

## Conflict of Interest

The authors declare that the research was conducted in the absence of any commercial or financial relationships that could be construed as a potential conflict of interest.

## Publisher's Note

All claims expressed in this article are solely those of the authors and do not necessarily represent those of their affiliated organizations, or those of the publisher, the editors and the reviewers. Any product that may be evaluated in this article, or claim that may be made by its manufacturer, is not guaranteed or endorsed by the publisher.

## Footnotes

^1^See http://www.doynefarmer.com/book.

^2^Personal correspondence with historian of science Lorraine Daston.
